# Mechanochemical synthesis of small organic molecules

**DOI:** 10.3762/bjoc.13.186

**Published:** 2017-09-11

**Authors:** Tapas Kumar Achar, Anima Bose, Prasenjit Mal

**Affiliations:** 1 School of Chemical Sciences, National Institute of Science Education and Research (NISER) Bhubaneswar, HBNI, P.O. Bhimpur-Padanpur, Via Jatni, Khurda 752050, Odisha, India

**Keywords:** ball-milling, green chemistry, mechanochemistry, solid-phase synthesis, solvent-free synthesis

## Abstract

With the growing interest in renewable energy and global warming, it is important to minimize the usage of hazardous chemicals in both academic and industrial research, elimination of waste, and possibly recycle them to obtain better results in greener fashion. The studies under the area of mechanochemistry which cover the grinding chemistry to ball milling, sonication, etc. are certainly of interest to the researchers working on the development of green methodologies. In this review, a collection of examples on recent developments in organic bond formation reactions like carbon–carbon (C–C), carbon–nitrogen (C–N), carbon–oxygen (C–O), carbon–halogen (C–X), etc. is documented. Mechanochemical syntheses of heterocyclic rings, multicomponent reactions and organometallic molecules including their catalytic applications are also highlighted.

## Introduction

The field of organic synthesis has experienced recently significant changes towards achieving the goal of more efficient and sustainable processes [1]. Thus, a new branch of chemistry termed as “Green Chemistry” has become a part of research interest by the chemists [2–4]. Green chemistry covers a wide range of research areas and generally deals with 12 principles [5–6] and few of them are: avoiding the use of volatile and toxic solvents, reducing the quantity of catalyst and reagents, using environmentally benign chemicals, atom-economical synthesis, minimization of chemical-waste/energy, etc. Non-conventional energy sources for chemical reactions such as microwave, mechanical mixing, visible-light and ultrasound are becoming surge of interest to the chemist as alternative energy sources in laboratories [7]. By imposing these techniques innumerable chemical transformations have been documented and thereby developing many existing protocols with superior results are further anticipated [8–9].

To address one of the major issues of green chemistry, i.e., minimizing chemical-waste/energy, solvent-free syntheses have become a popular research topic [8]. The mechanochemical techniques like ball-milling or hand grinding are considered to be promising candidates in solvent-free synthesis [10–11]. Mechanochemical methods deal with chemical transformations induced by mechanical energy, such as compression, shear, or friction [12]. Wilhelm Ostwald, a Russian-German chemist who received the Nobel Prize in 1909, mentioned the term “Mechanochemistry” as, like a branch of physical chemistry, i.e., thermochemistry, photochemistry and electrochemistry [13–14]. He defined the subject as “Mechanochemistry is a branch of chemistry which is concerned with chemical and physio-chemical changes of substances of all states of aggregation due to the influence of mechanical energy”. Moreover, according to IUPAC, a mechano-chemical reaction is a ‘Chemical reaction that is induced by the direct absorption of mechanical energy’ and with a note ‘Shearing, stretching, and grinding are typical methods for the mechano-chemical generation of reactive sites, usually macroradicals, in polymer chains that undergo mechano-chemical reactions’ [15].

The mechanistic understanding of mechanochemical reactions is still unclear [16]. A single idea could not be conceived because of the diversified nature of the reactions being practiced under mechanochemistry. Among the proposed models “hot spot” and “magma–plasma model” are mostly acceptable [17–18]. Other models like spherical model, dislocation and phonon theory, short-live-active center theory, kinetic and impulse model are also well known [19–20]. Nevertheless, this subject needs more attention to the both experimental and theoretical chemists [21].

The sophisticated technique of ball-milling or mechanomilling is the adaptation from the traditional grinding methods using a mortar and pestle. These mechanomillings methods are generally conducted in vibration mills or planetary mills at frequencies of 5–60 Hz [22–23]. The extensively used mechanomilling technique has limitations in controlling the reactions for air- and moisture-sensitive substances. In mechanomilling methods generally, the reactions are carried out in sealed vessels or jars of materials like stainless steel, tungsten carbide, zirconia, agate, etc. [24].

In the past decade, mechanochemical reactions were developed under the areas of chemistry like supramolecular chemistry [25–26], organic synthesis [27–28], nanoparticle synthesis, etc. [29–30]. The historical development of mechanochemistry [31], mechanistic aspects [32], mechanochemical synthesis of inorganic material [33], co-crystals [34], metal–ligand complexes [35], metal organic frameworks [36], polymers [37], etc. are well documented in seminal reviews and will not be discussed here. The organic mechanochemistry has remained undeveloped until the pioneering work reported by Toda in the 1980s [38] and Kaupp [24]. Due to several advantages, the area mechanochemistry has received significant attention over solution-based chemical methods and process developments [12,27,29]. The mechanochemical formation of carbon–carbon [39–40], carbon–heteroatom [41–42], metal–ligand coordination bonds [43], non-covalent interactions such as hydrogen bonds or π–π arene stacking interactions [44], etc. are popularly known in literature. In this review the efforts are given towards documentation of various mechanochemical reactions like organic bond formation reactions, multicomponent reactions, heterocyclic ring synthesis, synthesis of organometallic complexes and their catalytic applications, and so on.

## Review

### Mechanochemical organic synthesis

Famous philosopher Aristotle’s statement *“No Coopora nisi Fluida”* means ‘no reaction is possible in the absence of solvent’ and that was a common belief till last few decades. However, during the 1980s the pioneering works of Toda and co-workers proved that many organic reactions of solution chemistry would be reproducible in solid state too [22–23]. In the solid state reactions the ingredients are mixed to finely powdered form for better mixing. The ball-milling chemistry can better be conceived as the updated and sophisticated version of traditional grinding chemistry [38].

### Mechanochemical synthesis of C–C bond

More atom economic, energy efficient, time efficient and mild syntheses of C–C bonds are always desired. The solvent-free mechanomilling technique can also be an important alternative to replace traditional hand grinding methods [45]. Many solution-based C–C bond synthesis methods are reproducible under mechanomilling conditions with improved time and energy efficiency [46–47]. In this section some of the most important C–C bond forming reactions and their advantages are discussed.

#### Aldol reaction

**Scheme 1:**
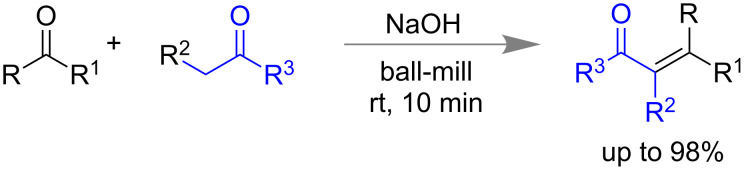
Mechanochemical aldol condensation reactions [48].

In 2000, Raston and Scott first reported the aldol condensation reaction using veratraldehyde, 4-phenylcyclohexanone and 1-indanone in the presence of NaOH in a vibrating ball mill and the products were obtained in the yield up to 98% within 10 min (Scheme 1) [48].

**Scheme 2:**
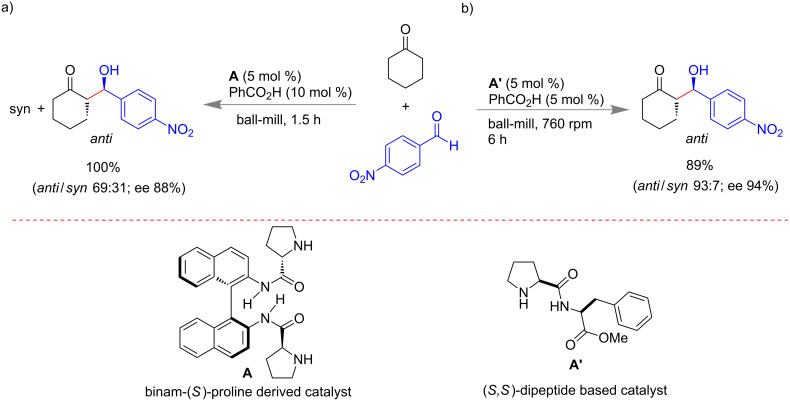
Enantioselective organocatalyzed aldol reactions under mechanomilling. a) Based on binam-(*S*)-proline derived catalyst [49]. b) Report using α,α-dipeptide-based catalyst [50].

However, the asymmetric version of a mechanochemical aldol condensation reaction was reported by Guillena and Nájera with co-workers (Scheme 2a) in 2008. Reactions between various ketones and aldehydes under solvent-free conditions were performed using a combination of (*S*)-binam-L-Pro (**A**, 5 mol %) and benzoic acid (10 mol %) as organocatalyst [49].

Juaristi and co-workers investigated the mechanistic aspects of α,α-dipeptide derivatives of a (*S*)-proline- (**A′**)-catalyzed asymmetric aldol reaction (Scheme 2b) under solvent-free mechanomilling [50]. By varying the electron density on the aromatic aldehydes, it was observed that electron deficient aldehydes provided a better yield with excellent stereo selectivity over electron rich systems. The observed result suggests that a π–π stacking interaction between electron-poor aromatic aldehydes and aromatic ring of the organocatalyst plays a crucial role for excellent yield and selectivity. Apparently the solvent-free system enhances the rigidity of the transition state for more selective reactions under mechanochemical activation.

#### Michael addition

**Scheme 3:**
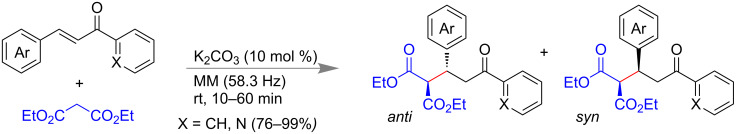
Mechanochemical Michael reaction [51].

Generally strong bases like NaOH, KOH, NaOEt etc. have been used as catalyst for the Michael addition of 1,3-dicarbonyl compounds to α,β-unsaturated ketones. In 2004, Wang and co-workers first reported a mechanochemical Michael reaction of 1,3-dicarbonyl compounds with chalcones and azachalcones using the mild base K_2_CO_3_ (Scheme 3). Michael adducts were isolated with good to excellent yield (76–99%) in a high-speed vibration mill (HSVM) within 10–60 min [51].

**Scheme 4:**
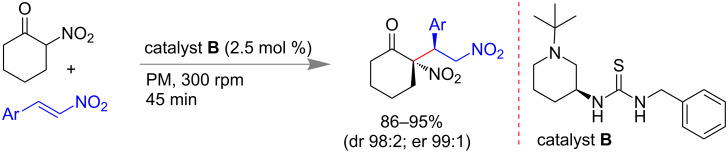
Mechanochemical organocatalytic asymmetric Michael reaction [52].

Bolm and co-workers reported an organocatalytic asymmetric version of Michael addition reaction under planetary-milling (PM) conditions. Differently substituted thiourea-based organocatalysts were screened for the reaction to achieve stereoselective adducts through hydrogen bonding. Only with 2.5 mol % of thiourea-based catalyst **B**, α-nitrocyclohexanone and nitroalkene derivatives could undergo a Michael addition to yield up to 95% of the desired product within 30 min (Scheme 4). Excellent stereoselectivity was also achieved with a diastereomeric ratio of 98:2 and enantiomeric ratio up to 99:1. Simple flash column chromatographic purification methods, low catalyst loading, gram scale synthesis, etc. were advantageous for the reaction [52].

#### Morita–Baylis–Hillman reaction

**Scheme 5:**
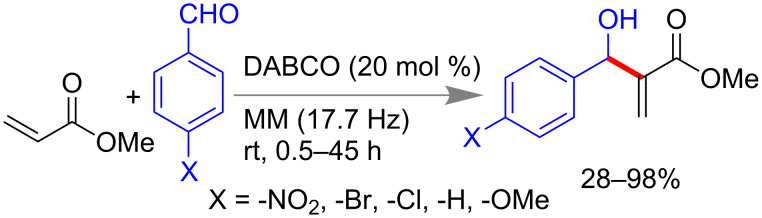
Mechanochemical Morita–Baylis–Hillman (MBH) reaction [53].

The Morita–Baylis–Hillman reaction (MBH) employs olefins, tertiary amine catalysts and electrophile aldehydes to produce multifunctional products. Mack et al., found a significant enhancement in the rate of a Morita–Baylis–Hillman (MBH) reaction under ball milling conditions (Scheme 5) compared to the conventional method that generally takes days to a week for completion. The reaction of methyl acrylate with different *para*-substituted aryl aldehydes in the presence of 20 mol % 1,4-diazabicyclo[2.2.2]octane (DABCO) catalyst at 0.5–45 h yielded the MBH products in 28–98% yield [53].

#### Wittig Reaction

**Scheme 6:**
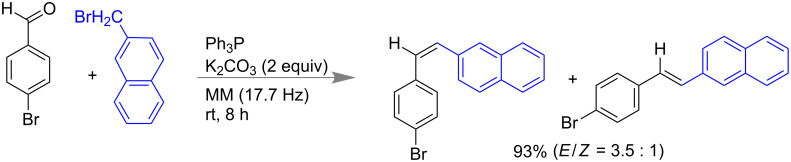
Mechanochemical Wittig reactions [55].

Pecharsky and co-workers reported the solvent-free mechanochemical synthesis of phosphonium salts [54] and phosphorus ylides [55] in the presence of the weak base K_2_CO_3_. Mechanochemically prepared phosphorous ylide from triphenylphosphine in presence of K_2_CO_3_ was utilized for a one-pot solvent-free Wittig reaction of organic halides with aldehydes or ketones (Scheme 6) [55].

#### Suzuki Coupling

**Scheme 7:**
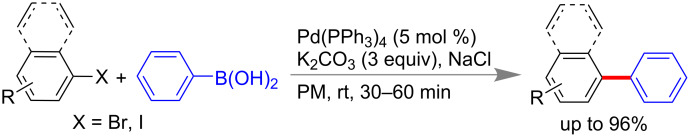
Mechanochemical Suzuki reaction [56].

In 2000, Peters and co-workers first reported the palladium-catalyzed Suzuki coupling reaction under ball-milling conditions [56]. In a planetary mill for 30–60 min, the mixture of aryl halide (1.0 equiv), phenylboronic acid (2.0 equiv), K_2_CO_3_ (3.0 equiv) and Pd(PPh_3_)_4_ (5 mol %) resulted in coupled products with 96% yield (Scheme 7). The NaCl was used as an additive to make the reaction mixture sufficiently powdery for uniform mixing.

**Scheme 8:**
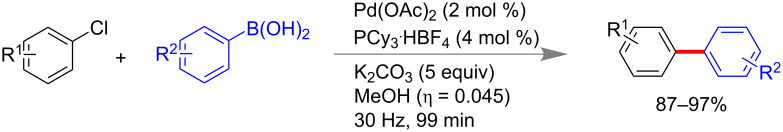
Mechanochemical Suzuki–Miyaura coupling by LAG [57].

The use of aryl chlorides is generally restricted in Suzuki reactions because of their low reactivity. Recently, Li and Su with co-workers have developed a liquid-assisted grinding (LAG) method for the Suzuki–Miyaura coupling between aryl chlorides and boronic acids to synthesize the biaryls in nearly quantitative yield. Under optimized conditions 2 mol % Pd(OAc)_2_ and 4 mol % of PCy_3_·HBF_4_ along with an excess K_2_CO_3_–MeOH led to biaryls within 99 min and with a yield up to 97% (Scheme 8) [57].

#### Heck reaction

**Scheme 9:**
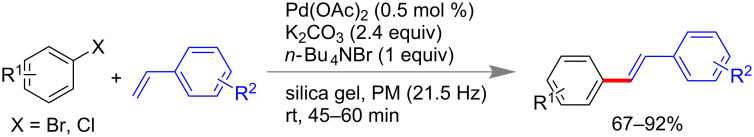
Mechanochemical Heck reaction [59].

Frejd and co-workers reported the first mechanochemical Heck reaction [58]. Su and co-workers demonstrated that (*E*)-stilbene derivatives were synthesized by the coupling of styrenes with aryl bromides or aryl chlorides (Scheme 9) [59].

#### Sonogashira reaction

**Scheme 10:**
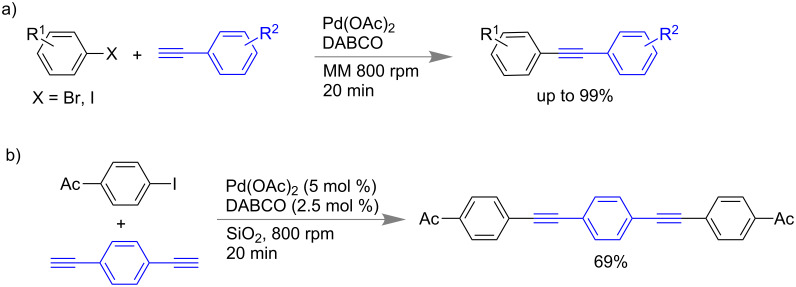
a) Sonogashira coupling under milling conditions. b) The representative example of a double Sonogashira reaction of *p*-iodoacetophenone with 1,4-bis-ethynyl benzene.

Stolle and co-workers have reported a Sonogashira coupling reaction under ball milling conditions in which the reactions were done in absence of any copper catalyst or any additional ligands [60]. In presence of palladium salts (Pd(OAc)_2_ or Pd(PPh_3_)_4_) and DABCO (1,4-diazabicyclo[2.2.2]octane) various acetylenes and aryl halides were coupled to obtain the Sonogashira coupling products in excellent yields (near quantitative, Scheme 10a). The reactions were reported for aliphatic alkynes as well. In Scheme 10b, an example of a double Sonogashira reaction is shown [60].

#### Oxidative cross-dehydrogenative coupling

**Scheme 11:**
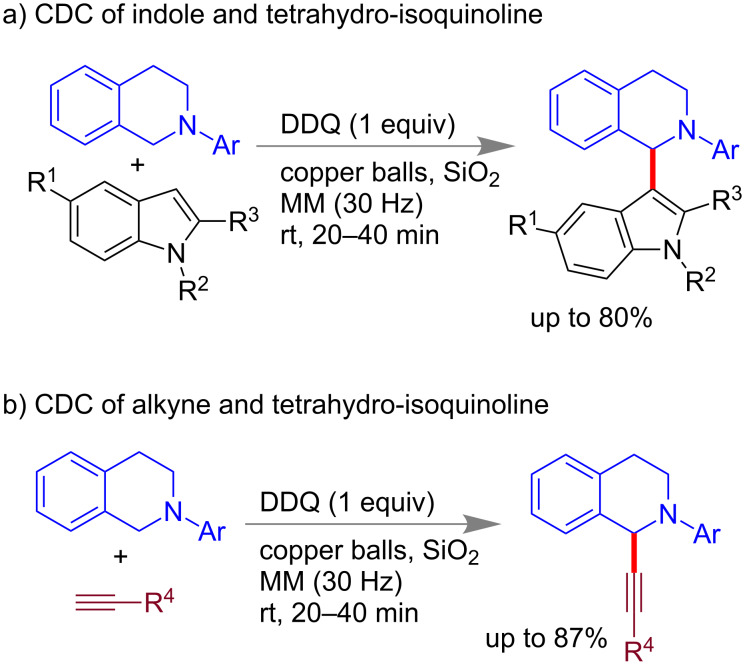
Copper-catalyzed CDC reaction under mechanomilling [67].

Copper-catalyzed mechanochemical oxidative cross-dehydrogenative coupling (CDC) reactions [61–66] of tetrahydroisoquinolines with alkynes and indoles was reported by Su and co-workers (Scheme 11) using 2,3-dichloro-5,6-dicyanoquinone (DDQ) as an efficient oxidant [67].

**Scheme 12:**
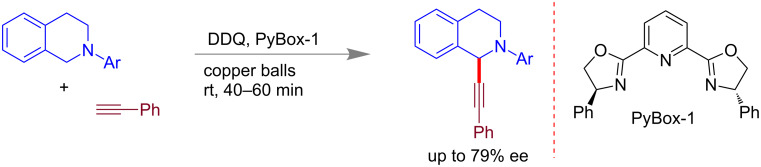
Asymmetric alkynylation of prochiral sp^3^ C–H bonds via CDC [68].

Su and co-workers have also reported an asymmetric version of the CDC reaction between terminal alkynes and sp^3^ C–H bonds under high speed ball milling conditions [68]. Several optically active 1-alkynyl tetrahydroisoquinoline derivatives were synthesized using a pyridine-based chiral ligand (PyBox, Scheme 12) in the presence of DDQ (2,3-dichloro-5,6-dicyano-1,4-benzoquinone). The coupling products were isolated in fair yields with ee’s (enantiomeric excesses) up to 79%. The milling copper balls were also identified as reacting catalyst.

**Scheme 13:**
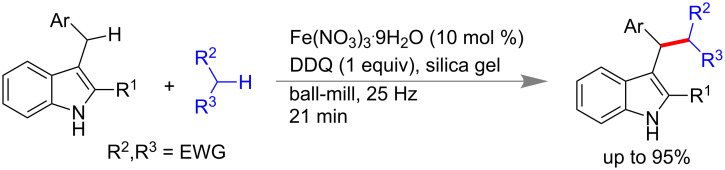
Fe(III)-catalyzed CDC coupling of 3-benzylindoles [69].

Su and co-workers reported an Fe(III)-catalyzed coupling of 3-benzyl indoles with molecules having active methylene group under solvent-free ball-mill in presence of silica gel as milling auxiliary. Using 10 mol % Fe(NO_3_)_3_·9H_2_O as catalyst and 1.0 equiv of DDQ afforded good yield of desired product at 25 Hz within 30 min (Scheme 13) [69]. The oxidant DDQ was added in portions at 7 min intervals to get better yields. Different active methylene compounds like diethylmalonate, dibenzylmalonate, malonitrile, and unsymmetrical 1,3-dicarbonyl compounds were explored for the CDC reaction.

**Scheme 14:**
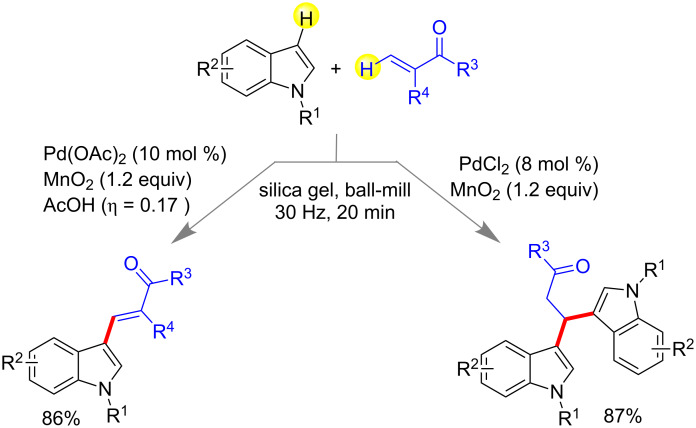
Mechanochemical synthesis of 3-vinylindoles and β,β-diindolylpropionates [70].

They have also demonstrated a mechanochemical synthesis of 3-vinylindoles and β,β-diindolylpropionates by C–H activation. Substituted indoles and ethyl acrylates were reacted in presence of 10 mol % of Pd(OAc)_2_ and 1.2 equiv of MnO_2_ to afford highly substituted 3-vinylindoles using silica gel and acetic acid (LAG). Contrastingly, when acrylic esters were treated with 8 mol % of PdCl_2_ and in absence of acetic acid, β,β-diindolylpropionates were obtained as the major product (Scheme 14) [70].

### C–N bond synthesis

**Scheme 15:**
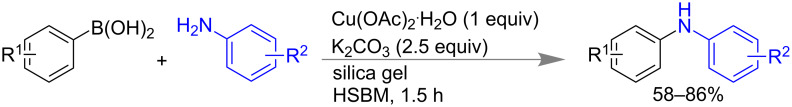
Mechanochemical C–N bond construction using anilines and arylboronic acids [78].

Amongst C–N bonds the amide bonds are most abundant and important too [71]. According to the American Chemical Society (ACS) and the Green Chemistry Institute (GCI), the “amide bond formation avoiding poor atom economy reagent” is one of the top challenges for organic chemists [72]. Easy, economical, selective and convenient approaches on C–N bond syntheses are of great importance [73–76]. In view of this, chemists have introduced alternative energy sources like, microwave, sonication, mechanomilling, etc. [12,27,77]. Su and co-workers reported a copper-catalyzed arylation of anilines using arylboronic acid under high speed ball-milling conditions. Using 1.0 equiv of Cu(OAc)_2_ and 2.5 equiv of K_2_CO_3_ and in the presence of milling auxiliary silica gel, differently substituted arylboronic acid and anilines led to *N*-arylated products in 58–86% yield (Scheme 15) [78]. They have also explored the reactivity of other amines like alkyl, primary, secondary, heterocyclic, etc.

**Scheme 16:**
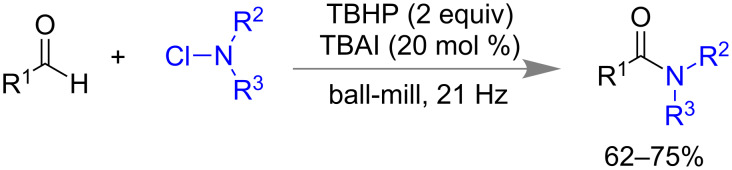
Mechanochemical amidation reaction from aromatic aldehydes and *N*-chloramine [79].

Mal and co-workers reported a metal free, solvent-free and room temperature synthesis of amide bonds at 62–75% yield under ball-milling (21 Hz) from aromatic aldehydes and *N*-chloramine in presence of 20 mol % of tetrabutylammonium iodide (TBAI) and 2.0 equiv of TBHP (Scheme 16) [79]. Aromatic aldehydes having electron-donating or -withdrawing substituents and different *N*-chloramines were well tolerated for this moderately yielding reaction.

**Scheme 17:**
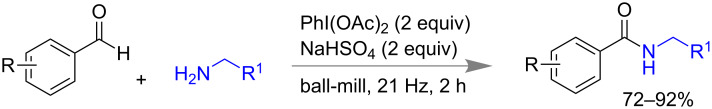
Mechanochemical CDC between benzaldehydes and benzyl amines [81].

They have also reported a method of controlling the chemical reactivity of contact explosives by exploiting weak interactions or soft force [80] during amide bond synthesis under ball-milling conditions. Cross dehydrogenative coupling reactions between benzaldehydes and benzylamines were performed in presence of phenyliodine diacetate (PIDA) using the acid salt NaHSO_4_ [81]. The highly exergonic reaction (contact explosive) of acidic iodine(III) and basic amines were safely controlled at maximum contacts (solvent-free) by the acid salt NaHSO_4_. Using 2.0 equiv of both NaHSO_4_ and PIDA, 72–92% of amides were isolated within 2 h (Scheme 17) [81].

**Scheme 18:**
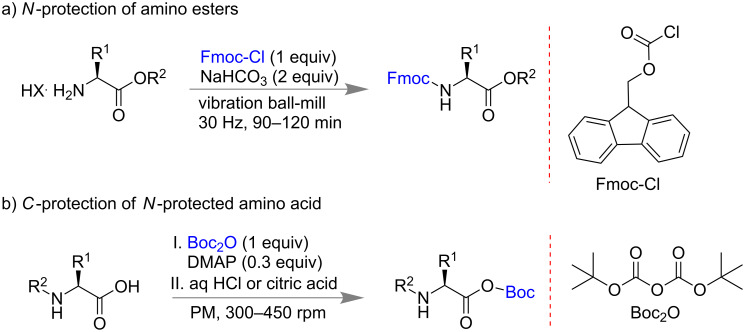
Mechanochemical protection of -NH_2_ and -COOH group of amino acids [85].

Amino acids are one of the important biomolecules for example as building block of peptides and proteins [75,82–84]. During the synthetic application of these molecules protection of -NH_2_ and -COOH group are needed. The traditional protection chemistry involves hazardous solvents, direct handling of corrosive reagents, longer reaction time, and tedious purification processes, etc. Therefore, methodologies involving mild reaction conditions, simple purification processes are always desirable. In 2014, Colacino and co-workers reported the protection of -NH_2_ and -COOH groups of amino acids by solvent-free milling methods using two different conditions [85]: 1) carbamoylation of amino esters using Fmoc-Cl and NaHCO_3_ (base); 2) esterification of *N*-protected amino acid using different dialkyl dicarbonate or alkyl chloroformate in the presence of DMAP as catalyst and followed by acidic workup. For *N*-terminal protection, different precursors like Fmoc-Cl, benzoyl chloride and Boc_2_O were used successfully to get nearly 90% yields for α-amino esters in 90–120 min (Scheme 18).

**Scheme 19:**
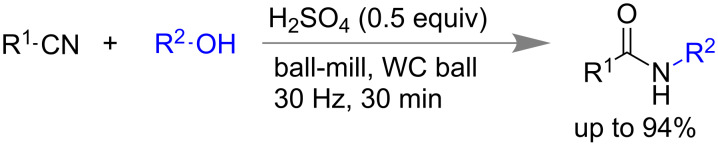
Mechanochemical Ritter reaction [87].

The Ritter reaction is another significant carbon–nitrogen (C–N) bond forming reaction in the synthesis of amides [86]. Generally, a nitrile and a tertiary alcohol in presence of a strong acid react to create amides. Major drawbacks associated with this method are the requirement of stoichiometric amounts of strong acid, higher temperature, narrower substrate scope, etc. In 2015, Gredičak and co-workers developed a milder version of the Ritter reaction under mechanomilling conditions. Using 0.5 equivalents of H_2_SO_4_, amides were isolated in good yields within 30 min of reaction time (Scheme 19) [87]. Various aromatic and aliphatic nitriles including acetonitrile, alcohols like *tert*-butanol and other secondary alcohols were used for this reaction. In case of solid nitriles 1.0 equiv of nitromethane was added during the grinding process to stabilize the carbocation species. This method was proved to be efficient by performing the reaction at 9.7 mmol scale to obtain 84% yield of the product.

### C–O bond formation reaction

**Scheme 20:**
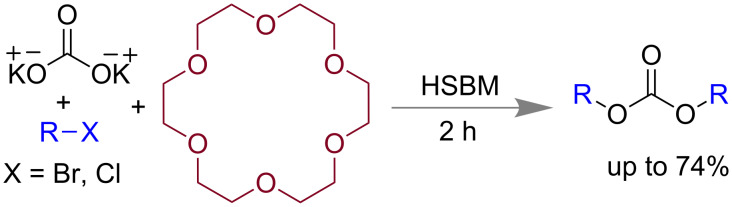
Mechanochemical synthesis of dialkyl carbonates [90].

Carbon–oxygen (C–O) bonds are widely present in molecules containing ester, carbamate and amino acid, etc. [88]. Traditional solution-based C–O bond synthesis generally needs large amount of solvents, excess chlorinating agent, harsh reaction conditions, a tedious isolation process, etc. compared to solvent-less grinding or mechanomilling [89]. In 2011, Mack and co-workers applied the high-speed ball milling (HSBM) technique for the synthesis of dialkyl carbonates [90]. Using potassium carbonate, alkyl halide and 2 equiv of phase-transfer catalyst 18-crown-6 yielded dialkyl carbonate in 74%. However, in absence of 18-crown-6 the yield was only 2% at 17 h (Scheme 20).

**Scheme 21:**
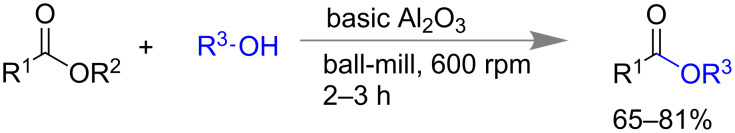
Mechanochemical transesterification reaction using basic Al_2_O_3_ [91].

Transesterification is a synthetic approach mostly being used for making higher homologous esters from the simpler ones. Ranu and co-workers developed simple method for transesterification under mechanomilling [91]. The mixture of ester and alcohols were adsorbed on the surface of basic alumina and followed by milling of the materials for 2–3 h led to 65–81% of trans-esterified product (Scheme 21). Differently substituted benzene rings including hetero-aromatics were also well tolerated under the similar condition.

**Scheme 22:**
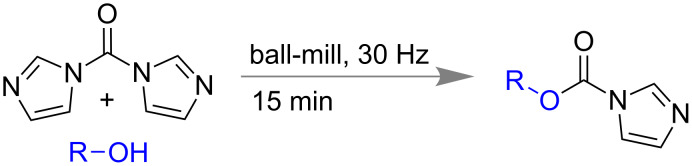
Mechanochemical carbamate synthesis [92].

Colacino and co-workers reported the preparation of carbamates by using 1,1’-carbonyldiimidazole (CDI) and in presence of either alcohols or amines as nucleophile [92]. When 2 equiv of CDI was treated with alcohol in a mixer mill at 30 Hz, within 15 min imidazolecarboxylic acid derivatives were isolated with a new C–O bond formation (Scheme 22).

### C–X bond forming reactions

**Scheme 23:**
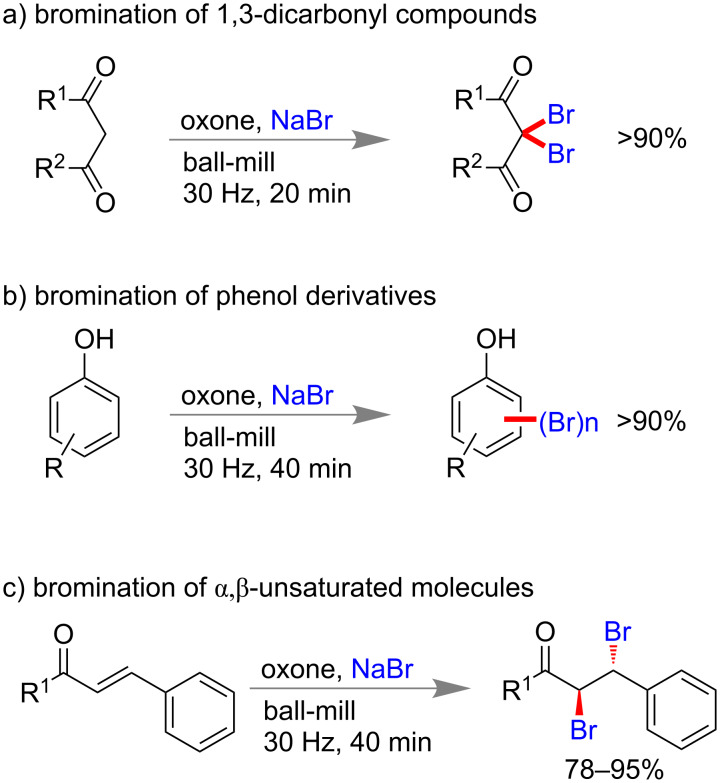
Mechanochemical bromination reaction using NaBr and oxone [96].

Carbon–halogen (C–X) bond forming reactions are also significant in organic synthesis because aryl halides are important synthons for the synthesis of many natural and non-natural products [93–94]. In 2005, Rahman and co-workers reported a pioneering solid state benzylic bromination of diquinoline derivatives via *N*-bromosuccinimide (NBS) [95]. In 2012, Wang and co-workers reported bromination of phenol derivatives, chalcones, 1,3-dicarbonyl compounds using NaBr as bromine source and oxone as oxidant under ball-milling conditions [96]. Within 1 h they could isolate more than 90% of mono or poly-brominated products of phenol and 1,3-dicarbonyl compounds (Scheme 23). α,β-Unsaturated carbonyl compounds could also undergo a *trans*-bromination reaction efficiently within 40 min.

**Scheme 24:**
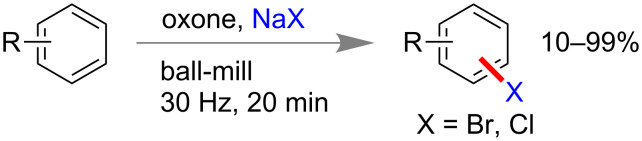
Mechanochemical aryl halogenation reactions using NaX and oxone [97].

Following to Wang’s report, Stolle and co-workers also reported a similar method of aryl bromination and chlorination using NaBr and NaCl, respectively, in the presence of oxidizing agent oxone (Scheme 24) [97].

**Scheme 25:**
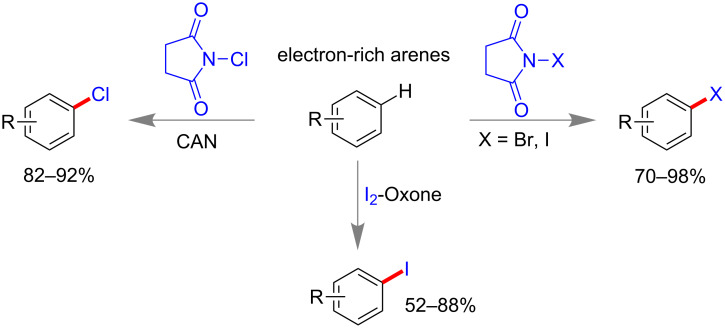
Mechanochemical halogenation reaction of electron-rich arenes [88,98].

Carbon–carbon double (C=C) and triple (C≡C) bonds-containing compounds are also reported to undergo dihalogenation reactions under mechanochemical conditions. In 2014, Mal and co-workers reported a mild aryl halogenation reaction using respective *N*-halosuccinimide (NXS) under solvent-free ball milling condition [88]. Aryl rings containing electron donating groups worked efficiently to yield 70–98% of mono or dibromo derivatives within 2 h. Similarly, NIS led to aryl iodination in near quantitative yield and NCS failed to produce any chlorination product (Scheme 25). However, NCS-cericammonium nitrate (CAN) successfully yielded mono-chlorinated products [88]. Consecutively, the same group reported metal-free oxidative iodination of electron rich aromatic rings with molecular iodine and oxone (Scheme 25) [98]. This method proved to be highly chemoselective and no benzylic iodination could be observed in case of alkyl benzenes. Interestingly, benzaldehyde derivatives did not lead to any over-oxidation to acids in presence of oxone.

**Scheme 26:**
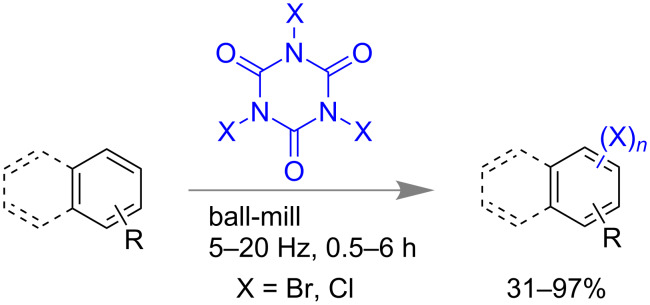
Mechanochemical aryl halogenation reaction using trihaloisocyanuric acids [100].

Trihaloisocyanuric acids are also used effectively for halogenations of arenes and 1,3-dicarbonyl compounds and double bond-containing systems [99]. Moorthy and co-workers investigated the potential of tribromoisocyanuric acid (TBCA) and trichloroisocyanuric acid (TCCA) under a solvent-free mechanomilling system for halogenations of electron rich arenes. The reactions were found to have yields above 80% for most of the cases but with poor selectivity in mono- or polybrominations (Scheme 26). They have also explored halogenations of 1,3-dicarbonyl compounds to obtain dihalo derivatives in excellent yield [100].

**Scheme 27:**
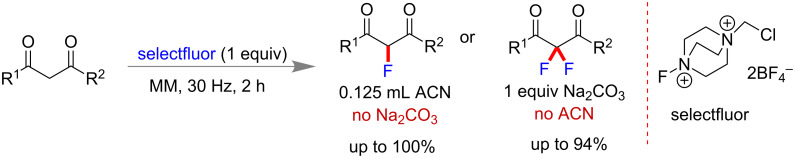
Mechanochemical fluorination reaction by LAG method [102].

In 2016, Browne and co-workers reported selective mechanochemical fluorination of 1,3-dicarbonyl compounds using selectfluor [101–102]. They could control the selectivity of the reaction through LAG using ACN (≈10% v/v of total materials) to get predominantly mono-fluorinated product over difluorinated derivatives (Scheme 27). Contrastingly, addition of 1.0 equiv of Na_2_CO_3_ led to switching of the selectivity predominantly towards di-fluorinated product [102].

### Multi-component reactions

**Scheme 28:**
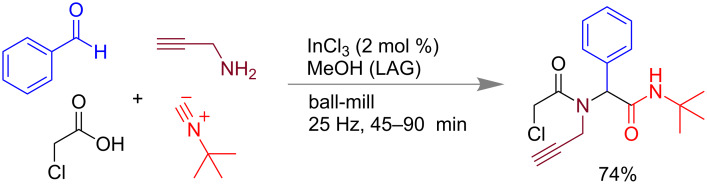
Mechanochemical Ugi reaction [116].

Multi-component reactions are one of the most powerful tools for the one pot synthesis of complex molecular structures with various functional groups [103–108]. Starting from the development of the Strecker synthesis of amino acids, many variations have been made till date. In solution these reactions generally proceed via a series of equilibrium processes and finally leading to the product through thermodynamic control [109–110]. However, in mechanochemical methods reactions are kinetically controlled [111]. Mechanochemical methods of the Mannich reaction, Paal–Knorr synthesis, Bigineli reaction, Hantzsch reaction, and syntheses of substituted pyran, thiophene, isoquinoline derivatives, etc. are also reported [104,107,112–113]. Isocyanide-based multi-component reactions are also well known [114–115]. Recently, in 2016 Juaristi and co-workers have reported Ugi 4-component reactions (4-CR) by liquid-assisted grinding (LAG) using MeOH. Equimolar amounts of benzaldehyde, chloroacetic acid, *tert-*butyl isocyanide, and propargylamine in the presence of 2 mol % InCl_3_, under ball-mill yielded the desired Ugi product in 74% yield (Scheme 28) [116].

**Scheme 29:**
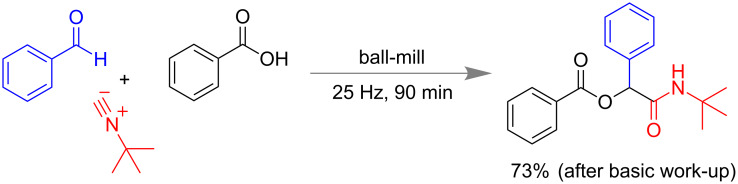
Mechanochemical Passerine reaction [116].

Juaristi and co-workers have also reported a mechanochemical Passerine 3-component reaction (3-CR). *tert-*Butyl isocyanide, benzaldehyde and benzoic acid in equimolar proportion under milling conditions for 90 min led to 73% of Passerine product (Scheme 29). Both electron-donating and -withdrawing substitutions on benzaldehydes or in benzoic acids have worked well under the mechano-chemical conditions [116].

**Scheme 30:**
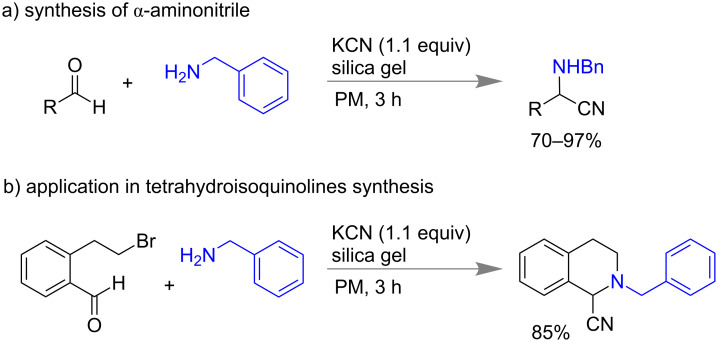
Mechanochemical synthesis of α-aminonitriles [120].

In a multicomponent Strecker reaction the syntheses of α-aminonitriles are generally done by condensation of aldehydes, ammonia and hydrogen cyanide [117–118]. The aminonitriles are important synthons for the preparation of nitrogen-containing heterocycles and amino acids [119]. In 2016, Bolm and co-workers reported a mechanochemical synthesis of α-aminonitriles using benzaldehyde, benzyl amine, KCN and the milling auxiliary SiO_2_ to isolate 70–97% of α*-*aminonitriles as the sole products. Contrastingly, in the solution of acetonitrile imines of benzaldehyde and amines were formed preferably. Different aromatic or heteroaromatic aldehydes including thiophene carboxaldehyde, pyridine carboxaldehyde and cyclohexyl carboxaldehyde as well as various amines like morpholine, aliphatic amines and sulfonamides worked smoothly under these conditions to obtain the desired product in 3 h. They have also extended the methodology for the synthesis of tetrahydroisoquinoline by using *o*-formyl phenethyl bromide with amine and KCN (Scheme 30) [120].

**Scheme 31:**
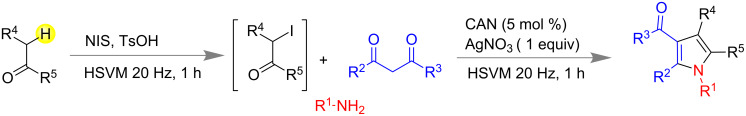
Mechanochemical Hantzsch pyrrole synthesis [121].

Since the discovery in 1890, the Hantzsch pyrrole synthesis is well known for the construction of poly substituted pyrroles [121–122]. In 1998, Jung and co-workers reported polymer supported solid phase synthesis of *N*-substituted pyrroles [123]. In 2013, Menendez and co-workers reported a ceric ammonium nitrate (CAN) and silver-nitrate-promoted three-component Hantzsch pyrrole synthesis under ball-milling conditions [121]. A ketone in presence of *N*-iodosuccinimide (NIS) and *p*-toluenesulfonic acid led to α-iodoketone in 1 h. Subsequent addition of the primary amine, β-dicarbonyl compound, 5 mol % CAN and 1 equiv silver nitrate led to the intermediate β-enaminone which further reacted with α-iodoketone following by a cyclo-condensation which resulted in the substituted pyrroles shown in Scheme 31.

**Scheme 32:**
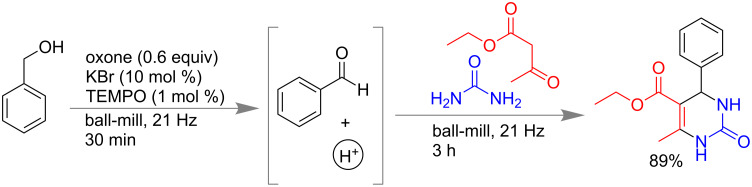
Mechanochemical Biginelli reaction by subcomponent synthesis approach [133].

The Biginelli reaction is a well-known 3-component reaction for the synthesis of dihydropyrimidinones [124–125]. During the last few decades many variations are adopted to improve the efficiency of this reaction for practical application towards drug discovery [126–128]. Modifications have been done in substrates by replacing urea with substituted ureas and thio urea, use of various 1,3-dicarbonyl compounds etc. Reactions using ionic liquids as reaction medium, solvent-free synthesis, microwave synthesis, use of different Lewis acids FeCl_3_, NiCl_2_, BiCl_3_, InBr_3_, use of Brønsted acids PTSA, etc. are also reported [129–130]. Recently, Mal and co-workers reported a mechanochemical Biginelli reaction by a subcomponent synthesis approach [131–133] in which the component aldehyde and catalytic amount of acid were generated in situ for the final step of dihydropyrimidinone synthesis. Benzyl alcohols were oxidized by a reagent combination of oxone (0.6 equiv), KBr (10 mol %) and 2,2,6,6-tetramethylpiperidin-1-yloxy radical (TEMPO, 1 mol %) to give benzaldehydes and H^+^ under solvent-free mechanochemical conditions within 30 min. Further, addition of 1,3-dicarbonyl compounds and urea derivatives within the same milling jar led to the desired products in 78–95% yield at 3 h (Scheme 32). Benzaldehydes with electron-donating or -withdrawing groups, heteroaromatic aldehydes, *N*-methyl urea and thio urea also resulted in good to excellent yield with high regioselectivity. It is interesting to note that the reaction was irreproducible in the solution of ethyl acetate at room temperature even after 24 h [133].

**Scheme 33:**
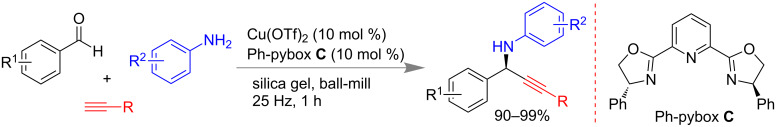
Mechanochemical asymmetric multicomponent reaction[134].

A mechanochemical asymmetric three component reaction is recently reported by Su and co-workers in the synthesis of propargyl amines using aldehyde, alkyne and amine under high vibration ball milling (HVBM) condition. Using 10 mol % of Cu(OTf)_2_ as catalyst, 10 mol % of Ph-PyBox ligand **C** and silica gel as milling auxiliary they could achieve near quantitative synthesis with >95% ee at 60 min (Scheme 33) [134]. However, aldehydes having strong electron-withdrawing or -donating groups yielded the product with lesser enantioselectivity. The silica-supported catalyst could be recovered from the reaction mixture by washing with DCM. They have also observed that an oven-dried catalyst worked effectively to give 99% of product with 99% ee up to few cycles.

### Heterocycle synthesis

**Scheme 34:**
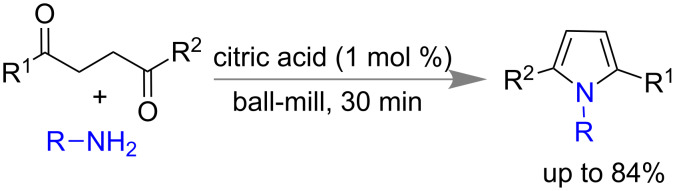
Mechanochemical Paal–Knorr pyrrole synthesis [142].

Multicomponent reactions [135], cyclo-condensations and cascaded transformations are common strategies to make heterocyclic ring [113] systems like pyrroles, pyrans, benzimidazoles, pyrimidines, indoles, etc. [114,136–139]. Further improvements are in demand for the development of synthesis with solvent-less, time efficient, less byproducts, energy saving, easy handling procedures, etc. [112,140–141]. In 2016, Rousseau and co-workers reported a solvent-free mechanochemical Paal–Knorr pyrrole synthesis using a solid bio-sourced acid like citric acid. Using substituted aniline, benzyl or aliphatic amine and 1,4-diketo compounds in presence of 1 mol % citric acid under ball-milling afforded the desired *N*-substituted pyrrole with quantitative yield (Scheme 34) [142].

**Scheme 35:**
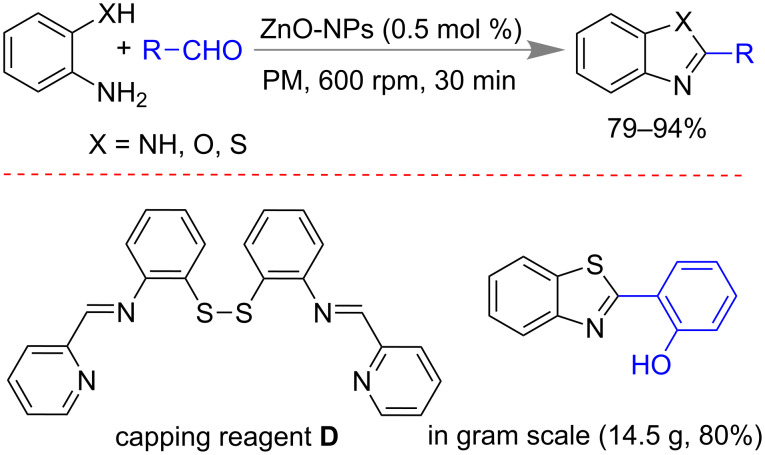
Mechanochemical synthesis of benzothiazole using ZnO nano particles [146].

Jang and co-workers reported a mechanochemical synthesis of benzimidazoles [143–144], benzoxazole [145] and benzothiazole derivatives in presence of ZnO nano particles as catalyst [146]. Using 0.5 mol % of ZnO nano particles which were grown on aromatic imine **D** as capping agent, resulted in the best yield within 30 min at 600 rpm. Differently substituted diamines, 2-aminothiophenol and 2-aminophenols reacted with benzaldehyde or aliphatic aldehyde derivatives to give 79–94% of the desired product (Scheme 35). Major advantage of this method was the regeneration of catalyst by filtration and washing with methanol. Secondly, the method was also applicable up to 10 g of 2-aminothiophenol and avoided the use of toxic metals which are common in benzimidazole synthesis [147–148].

**Scheme 36:**
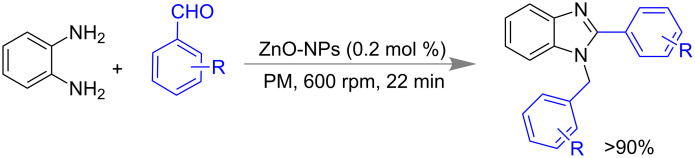
Mechanochemical synthesis of 1,2-di-substituted benzimidazoles [149].

Subsequently, the same group reported the preparation of 1,2-disubstituted benzimidazoles via mechanochemical activation using carboxymethylimidazole-based ionic-liquid-coated ZnO nano particles as catalyst (Scheme 36) [149]. The catalyst worked efficiently till to the fifth cycle after regeneration by filtration of product and washing with methanol. The method was scalable up to using of 8 g of *o*-phenylene diamine.

**Scheme 37:**
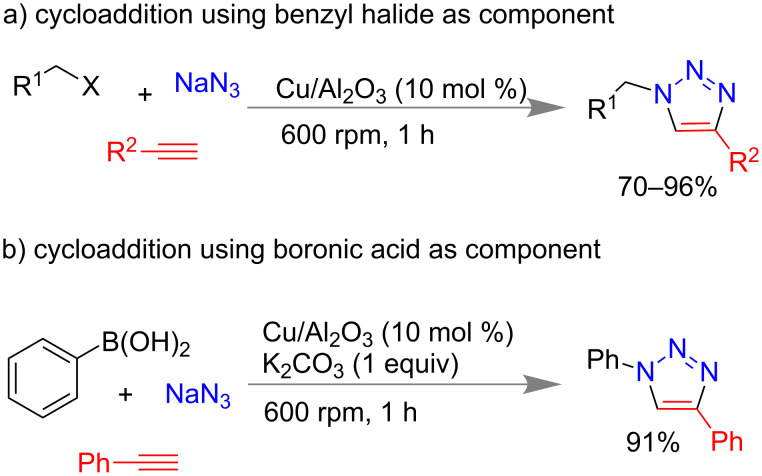
Mechanochemical click reaction using an alumina-supported Cu-catalyst [152].

1,2,3-Triazoles have important applications in pharmaceutical chemistry [150] and traditionally they are prepared by 1,3-dipolar cycloaddition reactions at high temperature, long reaction times and produce low yield with multiple products [151]. In 2013, Ranu and co-workers reported mechanochemical synthesis of triazole moiety (Scheme 37a) using benzyl halides, sodium azide and a terminal alkyne via an alumina-supported copper catalyst. Using 10 mol % of Cu/Al_2_O_3_, differently substituted phenyl acetylenes and aliphatic alkynes led to 70–96% yield of triazoles [152]. Phenyl boronic acids were also used to synthesize the triazole rings with additional 1 equiv of K_2_CO_3_ which resulted in >85% of product (Scheme 37b).

**Scheme 38:**
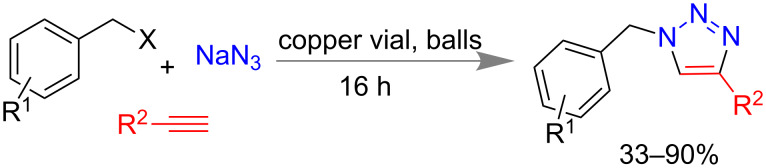
Mechanochemical click reaction using copper vial [155].

Mack and co-workers reported another mechanochemical variation of “click” reaction [153–154] where they could isolate 33–90% of triazole derivatives using copper reaction vial in ball mill for 16 h (Scheme 38). The same method was easily applicable to the synthesis using alkyl azide in 15 min [155].

**Scheme 39:**
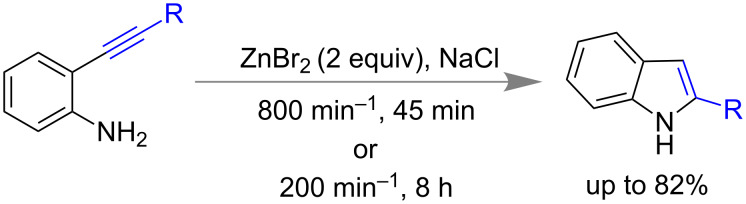
Mechanochemical indole synthesis [157].

Among various synthetic routes of indoles synthesis, Larock method [156] possibly be the important one which utilizes 2-alkynylaniline as intermediate towards intra-molecular cyclization. Stolle and co-workers also demonstrated mechanochemical synthesis of indoles using stoichiometric amounts of ZnBr_2_ and NaCl as milling auxiliary starting from 2-alkynylaniline derivatives (Scheme 39) [157]. They have correlated the milling frequency and time of reaction to the product yields and selectivity. For example, a) at higher frequency (800 min^−1^) for 45 min lower yield with less selectivity was observed and b) using lower frequency, 200 min^−1^ for 8 h led to 82% of yield with high selectivity.

**Scheme 40:**
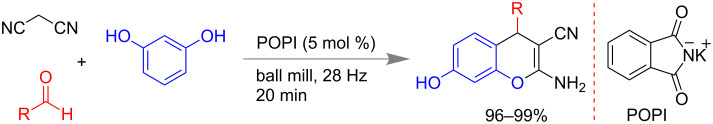
Mechanochemical synthesis of chromene [158].

In the traditional method of pyran synthesis the use of transition metal catalyst, corrosive acid, longer reaction time, hazardous organic solvent, and tedious isolation procedure are implemented. Dekamin and co-workers have demonstrated the synthesis of pyrans using potassium phthalimide (POPI) as a catalyst under ball-milling which is found to be advantageous over solution phase synthesis [158]. Malonitrile, benzaldehydes and electron-rich phenols in presence of 5 mol % of POPI, afforded near quantitative yield of chromene derivatives within 20 min (Scheme 40). Similarly, various benzaldehydes with electron-withdrawing groups at the *o*/*p*-position accelerated the reaction and electron-donating groups slowed that down. Hetero aromatic aldehydes also worked efficiently to give the products in 96–98% yield [158].

**Scheme 41:**
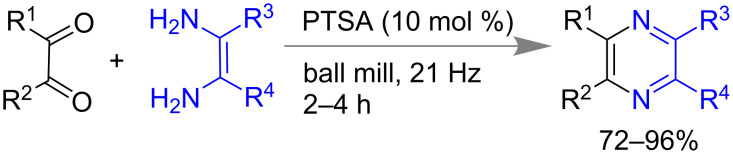
Mechanochemical synthesis of azacenes [169].

Acenes and hetero-acenes have important applications in material development such as semiconductors, photovoltaic cells, field effect transistors, organic light emitting diodes, etc. [159–165]. Moreover, the literature known methods adopted mainly harsh reaction condition and they are generally found to be low yielding [166–168]. Recently, Mal and co-workers reported mechanochemical synthesis of hetero-acenes from 1,2-dicarbonyl compounds and 1,2-diaminoarenes using 10 mol % *p*-toluenesulfonic acid as catalyst. Using this process they could isolate 72–96% of pyrazaacene, phenazine, bis(phenazine), bis(quinoxaine) derivatives (Scheme 41). Major advantages of this mechanomilling methods were time efficient (2–4 h), simple purification procedure (washing with polar solvent), high yielding, room temperature conditions, etc. Previously reported solvent-based synthesis required reflux for 3 days to get 30–40% yield [169].

### Miscellaneous bond formation reaction

#### Carbon–phosphorus bond synthesis

**Scheme 42:**
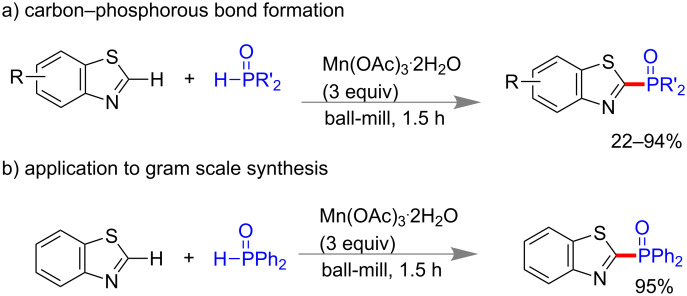
Mechanochemical oxidative C-P bond formation [170].

Recently, Wang and co-workers reported the first carbon–phosphorous (C–P) bond synthesis under mechanochemical conditions. Phosphonylation of benzothiazole and thiazole derivatives were done with organophosphorus compounds using 3 equiv of Mn(OAc)_3_·2H_2_O in a mixer mill for 1.5 h. Benzothiazole or thiazole rings having electron-donating or -withdrawing groups worked efficiently under this protocol. Different organophosphorus compounds including phosphine oxides, phosphinate ester, and phosphonate diester underwent C–P bond formation to give 22–94% of yield (Scheme 42). This method was also found to be applicable in gram scale synthesis with excellent yield. Mechanistically they have shown that the reaction followed a radical pathway [170].

#### C–Chalcogen bond formation

**Scheme 43:**
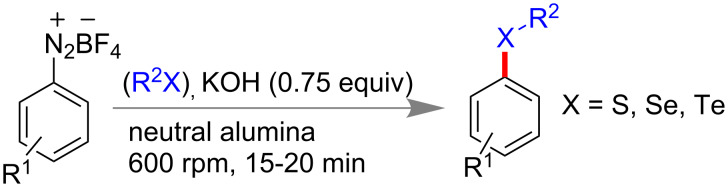
Mechanochemical C–chalcogen bond formation [171].

Ranu and co-workers reported carbon–chalcogen (C–S, C–Se, C–Te) bond formation from aryldiazonium tetrafluoroborate (1 equiv), diaryl chalocogenide (0.5 equiv) in a stainless steel jar at 600 rpm for 15 min. They have used KOH as base, neutral alumina as milling auxiliary. Both electron-donating and -withdrawing diazonium salts worked efficiently to give 70–90% of the products (Scheme 43) [171]. This solvent-free mechanomilling strategy reported to be superior to any solution phase synthesis because it avoids transition metals, could be performed in shorter reaction time and uses stable dichalcogenides rather than toxic thiols and selenols.

### Organometallic synthesis and catalytic application

#### Mechano-synthesis of organometallic compounds

**Scheme 44:**
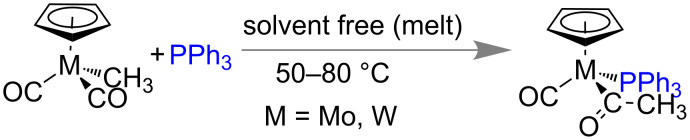
Solvent-free synthesis of an organometallic complex.

The last decade has witnessed a rapid growth of mechanochemistry in organic synthesis as well as in inorganic coordination chemistry [172]. However, the mechanochemical organometallic synthesis is still in its infancy due to certain difficulties under solvent-free synthesis. Recently the solid state syntheses of organometallic compounds have become popular. In their pioneering work Coville and co-workers presented solvent-free organometallic transformations (e.g., migratory insertion and ligand substitution reactions) at elevated temperature (Scheme 44) which have close resemblance to mechanochemistry [173].

**Scheme 45:**
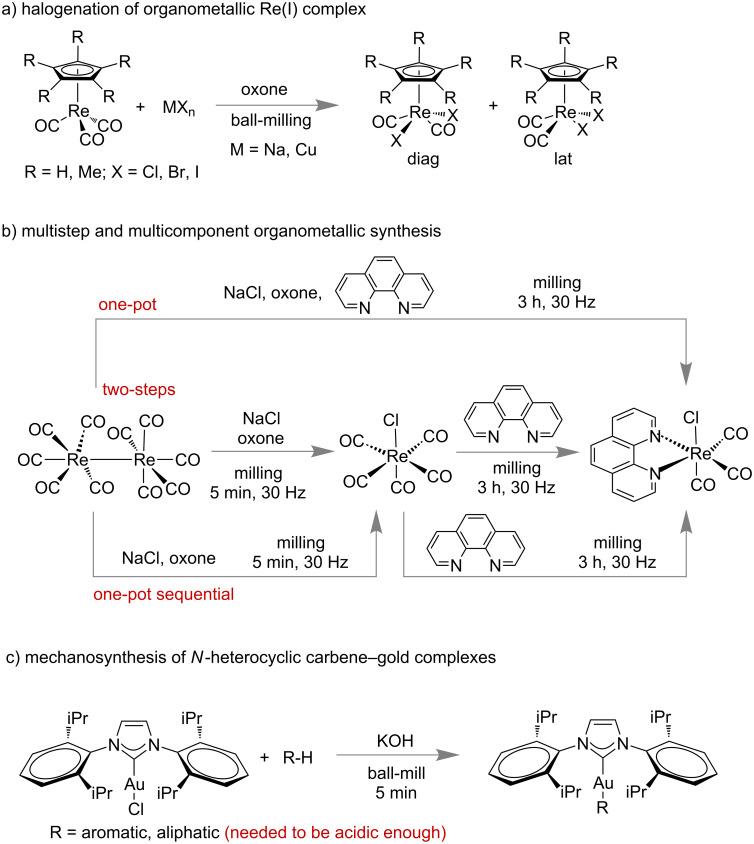
Selective examples of mechano-synthesis of organometallic complexes. a) Halogenation reaction of Re-complexes [175]. b) Multistep and multicomponent synthesis of Re-complexes [176]. c) Mechano-synthesis of NHC-Au complex [177].

The examples of mechanochemical organometallic complex synthesis are relatively small, but experienced significant growth in recent times. In the early 1990s, the first examples of mechanochemical organometallic reactions were discovered, included the synthesis of various indenyl, cyclopentadienyl and metallocarborane complexes [174]. In Scheme 45, few examples of mechanosynthesis of organometallic complexes are shown.

**Scheme 46:**
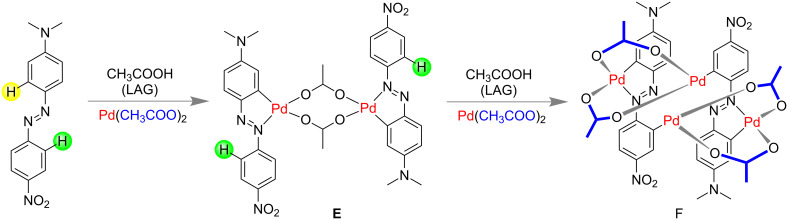
Mechanochemical activation of C–H bond of unsymmetrical azobenzene [178].

Ćurić and co-workers reported the first mechanochemical activation of a C–H bond of unsymmetrical azobenzene with Pd(OAc)_2_ [178]. The cyclopalladation process was highly regioselective and the rate of palladation was also faster than traditional solution phase processes. 4'-(*N,N*-dimethylamino)-4-nitroazobenzene with an equimolar amount of Pd(OAc)_2_ and 25 μL of glacial acetic acid (for LAG) resulted in regioselective C–H activation to give cyclopalladated complex **E** in 4.5 h where two Pd- and two azobenzene groups were involved. Treating this complex with another 1 equiv of Pd(OAc)_2_ resulted in a second C–H activation to give dicyclopalladated complex **F** in 7.5 h (Scheme 46). It is notable that the monocyclopalladated complexation generally takes 3 days in solution and dicyclopalladated complex in solution was never been identified [178].

**Scheme 47:**
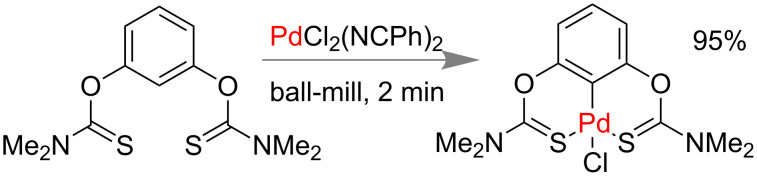
Mechanochemical synthesis of organometallic pincer complex [179].

Recently Aleksanyan and co-workers reported the first gram-scale synthesis of a Pd^II^ organometallic pincer complex under mechanomilling via C–H bond activation. After successful isolation of the Pd^II^ pincer complex by grinding of bis(thiocarbamate) and PdCl_2_(NCPh)_2_ they could scale up the reaction up to 1.76 mmol. Using a stainless steel jar they could isolate 95% of the pure pincer complex within 2 min (Scheme 47) [179].

**Scheme 48:**
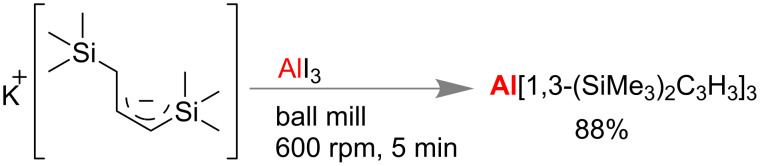
Mechanochemical synthesis of tris(allyl)aluminum complex [180].

Hanusa and co-workers developed a base-free mechanochemical synthesis of a tris(allyl)aluminum complex. Importantly, unsolvated tris(allyl)aluminum was never been isolated from solution, but mechanochemically found to be a high yielding reaction when bulky 1,3-bis(trimethylsilyl)allyl anion (Scheme 48) was reacted with aluminum iodide [180].

#### Catalytic application

**Scheme 49:**
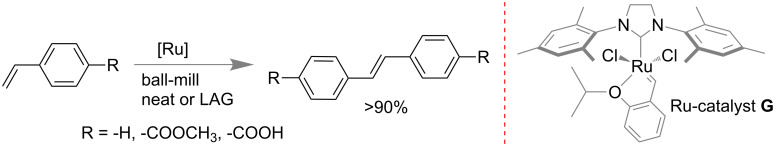
Mechanochemical Ru-catalyzed olefin metathesis reaction [181].

The success of the mechanochemical synthesis over traditional solvent-based synthesis in different areas has been recognized over the decades. Importantly catalytic application of these mechano-synthesized complexes are also explored. Friščić and co-workers recently reported an efficient mechanochemical approach towards Ru-based Hoveyda–Grubbs catalyzed olefin metathesis, cross-metathesis and ring-closing metathesis reactions (Scheme 49) [181]. Advantageously this methodology was applicable for both solid and liquid olefins.

### Mechanochemical C–H functionalization

**Scheme 50:**
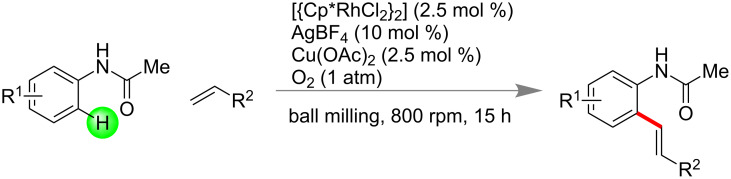
Rhodium(III)-catalyzed C–H bond functionalization under mechanochemical conditions [182].

Transition-metal-catalyzed activation and functionalization of inert C–H bonds of organic molecules provides a broad avenue in the synthesis of wide range of compounds. In 2015, Bolm and co-workers have successfully demonstrated rhodium(III)-catalyzed C–H bond functionalization under mechanochemical conditions [182]. Advantageously, the developed method adopted mild reaction conditions, i.e., in solvent-free medium and at room temperature. It required a minimum amount of toxic metal salt of Rh, Cu(OAc)_2_ as a redox modulator and dioxygen as a terminal oxidant (Scheme 50). This efficient technique was turned out to be a greener alternative to the common and mechanistically similar solution based method.

**Scheme 51:**
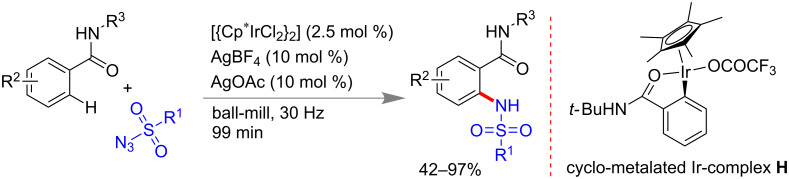
Mechanochemical Csp^2^–H bond amidation using Ir(III) catalyst [183].

They have also extended mechanochemical C–H functionalization methodology by varying the metal catalyst from rhodium to iridium. In 2016, using an Ir(III) catalyst an unprecedented *ortho*-selective Csp^2^–H bond amidation of benzamides with sulfonyl azides as the amide source was done under solvent-free ball mill conditions (Scheme 51) [183]. They could also isolate cyclic iridium complex **H** in ball-milling conditions.

**Scheme 52:**
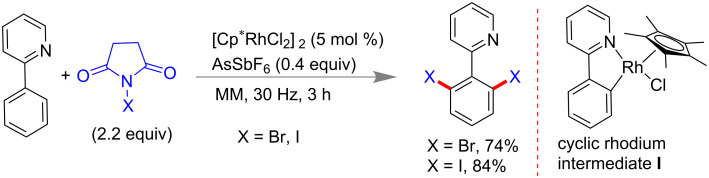
Mechanochemical Rh-catalyzed C_sp2_–X bond formation [184].

In 2015, the Bolm group reported the synthesis of [Cp*RhCl_2_]_2_ under LAG from rhodium(III) chloride hydrate and pentamethylcyclopentadiene (Cp*H) at lesser reaction time than solution-based protocols. Subsequently, they utilized the [Cp*RhCl_2_]_2_ for the solvent-free mechanochemical C–H bond functionalization of 2-phenylpyridine (Scheme 52). With 2.2 equiv of NXS (X = Br, I) and 5 mol % of [Cp*RhCl_2_]_2_ catalyst in a mixer mill, 74% and 84% of dibromo- and diiodo derivatives of 2-phenylpyridine, respectively, were isolated within 3 h [184].

**Scheme 53:**
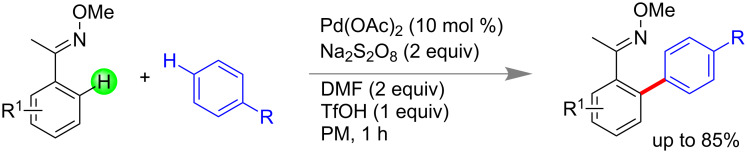
Mechanochemical Pd-catalyzed C–H activation [185].

Xu and co-workers developed a palladium-catalyzed site selective mechanochemical dehydrogenative C–H/C–H arylation between oxime and arene moiety for the construction of C_sp2_–C_sp2_ bond with high *para*-selectivity of arene component via LAG. Using 10 mol % of Pd(OAc)_2_, 2.0 equiv of Na_2_S_2_O_8_ and 1.0 equiv TfOH the biaryls were synthesized in good to excellent yield within 1 h. Dimethyl formamide (DMF) acted as ligand during the activation process (Scheme 53). The protocol was also equally applicable to electron deficient oximes and electron rich anilides [185].

**Scheme 54:**
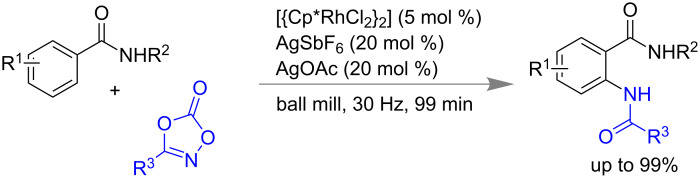
Mechanochemical Csp^2^–H bond amidation using Rh catalyst.

Bolm and co-workers reported a Rh-catalyzed amidation of Csp^2^–H bonds using dioxazolone as the amide source under ball milling conditions (Scheme 54). Using 5 mol % of Rh catalyst, 20 mol % of AgSbF_6_ and 20 mol % of AgOAc they have successfully achieved up to 99% of *ortho-*amidation product with diversely substituted arene moiety [186].

**Scheme 55:**
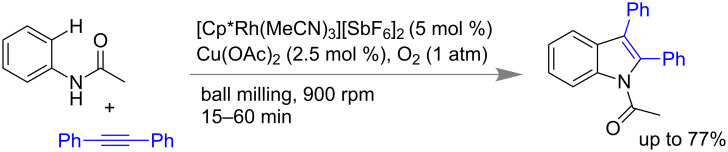
Mechanochemical synthesis of indoles using Rh catalyst [187].

Recently Bolm and co-workers developed a mechanochemical synthesis of an indole moiety via a Rh-catalyzed C–H functionalization strategy under planetary ball mill [187]. Using acetanilide and diphenylacetylene as the alkyne component in presence of 5 mol % Rh catalyst and 2.5 mol % Cu(OAc)_2_ and 1 atm O_2_ as terminal oxidant they could isolate up to 77% of differently substituted indole derivatives (Scheme 55).

### Advantages and limitations

**Scheme 56:**
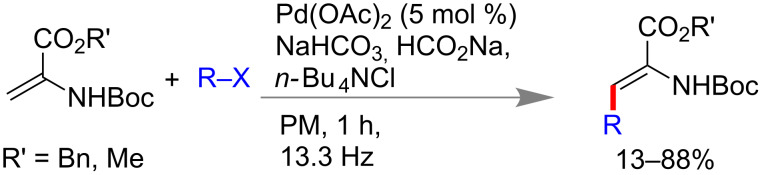
Mizoroki–Heck reaction of aminoacrylates with aryl halide in a ball-mill [58].

Over a couple of decades the area of mechanochemistry considered to be one of the best solvent-free synthetic methods. This area has become significantly interesting to chemists due to its benefits over conventional solution-based protocols. Importantly in mechanochemistry, avoiding traditional work-up might be considered as one of the major beneficial aspects. This benefit also leading to a significant development to green processes, turned out to be economical, time-efficient and environmentally benign. Easy purification procedures, towards quantitative conversion and minimum byproducts are additionally considered to be major significance to this method. Tullberg et al. investigated the Mizoroki–Heck reaction between iodobenzene and the methyl ester of *N*-Boc-protected aminoacylate under different conditions of energy (Scheme 56) and showed that efficiency under mechanomilling is far better over other methods [58].

**Scheme 57:**
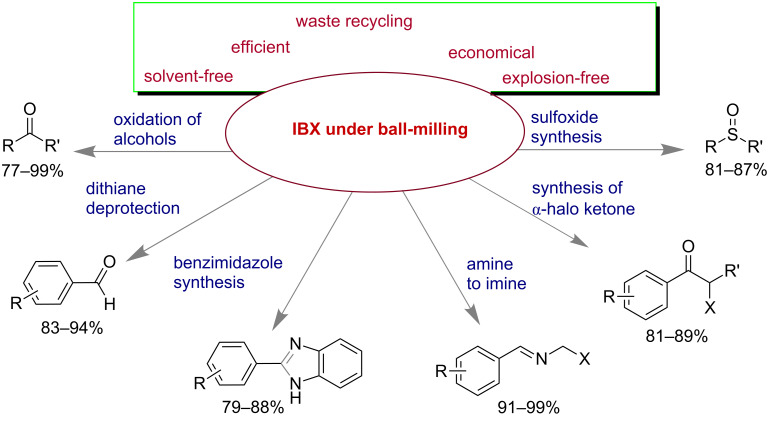
IBX under mechanomilling conditions [8].

Mal and co-workers have addressed the efficiency of 2-iodoxybenzoic acid (IBX) under mechanomilling conditions (Scheme 57) [8]. Generally the major drawback of IBX is its insolubility in common organic solvents except DMSO and also its explosive nature at higher temperature [188]. They could overcome these limitations by using IBX under solvent-free mechanomilling conditions. They have demonstrated various oxidation reactions, synthesis of benzimidazoles, deprotection of dithianes, etc. The byproduct iodosobenzoic acid (IBA) was recycled over 15 cycles with the help of the oxidant oxone. The economic benefits of IBX under ball milling was also discussed by comparing the literature-known DMSO mediated procedure [8].

**Scheme 58:**
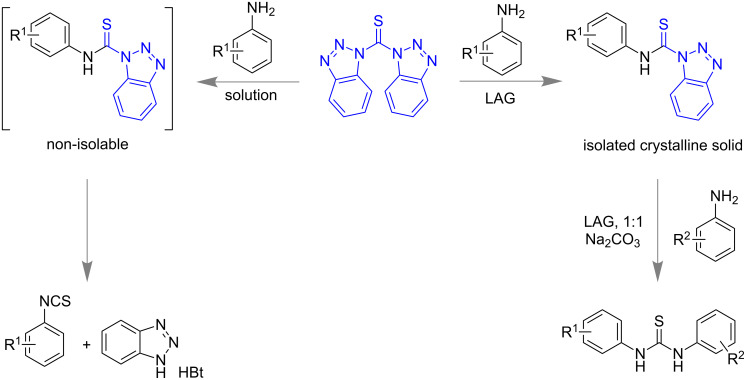
Thiocarbamoylation of anilines; trapping of reactive aryl-*N*-thiocarbamoylbenzotriazole intermediate in mechanochemical synthesis while not isolable in solution-based synthesis [190].

The bis(benzotriazolyl)methanethione-assisted thiocarbamoylation of anilines proceed through the formation of unisolable reactive intermediate, aryl *N*-thiocarbamoylbenzotriazole, which rapidly decomposes to the corresponding isothiocyanate in organic solvent [189]. The Štrukil and Friščić group successfully demonstrated the formation of aryl-*N*-thiocarbamoylbenzotriazole under the LAG (liquid-assisted grinding) synthesis (Scheme 58) [190]. Initially, in situ monitoring of mechanochemical thiocarbamoylation suggests the formation of reactive intermediate which gradually disappears with the formation of thiocarbamoylated product. Furthermore isolation and spectroscopic characterization of aryl-*N*-thiocarbamoylbenzotriazole intermediate clearly established the advantage of mechanochemistry over traditional solution-based synthesis and unwraps a new avenue for the mechanistic study as a promising technique.

Recently, the field of C–H activation has gained huge attention of chemists. It allows selective functionalization of C–H bonds to C–hetero atoms as discussed herein. Moreover, the major drawbacks are involvement of harsh reaction conditions like high temperature, longer reaction time, and huge amount of toxic organic solvents and handling of sensitive metal catalyst. But fortunately, mechanochemistry has overcome all these limitations and proved to be advantageous since it uses minimum amount of solvents, shorter reaction time, and easy handling of reagents and room temperature conditions. Despite the advantages of ball milling in chemical synthesis still there are some limitations to be noted. Mechanochemical methods are generally uncontrollable to temperature controlled reactions, time controlled reactions, in handling low boiling liquids, moisture sensitive systems, heterogeneous reactions, pressure controlled reactions, etc. The mechanochemistry is focused on making the known solution-based synthetic procedures more environmentally friendly by avoiding the solvent which is also one of the major drawbacks. So development of innovative bond formation reactions under mechanomilling should be highly appreciated that are inaccessible from solution phase chemistry.

## Conclusion

Significant progress has been made under the area of mechanochemistry during the last few decades owing to their improvement of environmentally sustainable and more selective processes. The major focus of this review is to cover the application of mechanochemistry in the synthesis of small organic molecules including heterocycles. In addition, the mechanosynthesis of organometallics as well as their selective applications in catalysis are also discussed. The understanding of the mechanism of mechanochemical reactions is still unclear and requires significant advancement in this research area. Improvement in new synthetic methodologies under mechanomilling conditions with better results are always demanding, rather than “greening” the solution phase synthesis.
